# Isolation and Plant Growth Promotion Effect of Endophytic Siderophore-Producing Bacteria: A Study on Halophyte *Sesuvium portulacastrum*

**DOI:** 10.3390/plants13192703

**Published:** 2024-09-27

**Authors:** Xinyi Cen, Hua Li, Yanhua Zhang, Lingfeng Huang, Yuanrong Luo

**Affiliations:** 1 Key Laboratory of the Ministry of Education for Coastal and Wetland Ecosystems, Xiamen University, Xiamen 361102, China; 2 Xiamen City Key Laboratory of Urban Sea Ecological Conservation and Restoration (USER), Xiamen University, Xiamen 361102, China; 3 College of the Environment and Ecology, Xiamen University, Xiamen 361102, China

**Keywords:** *Sesuvium portulacastrum*, root microbiome, endophytes, plant growth-promoting rhizobacteria (PGPR), siderophore

## Abstract

The objective of the present study was to isolate endophytes from the roots of the halophyte *Sesuvium portulacastrum*, which is applied for aquatic phytoremediation. From these endophytes, siderophore-producing bacteria were specifically isolated for their potential capacity to promote plant growth. The siderophore production capacity of the isolated bacteria was quantified, and a high-yield siderophore-producing strain was selected for further investigation. A total of 33 endophytic bacteria were successfully isolated and identified using a culturable approach. Of these, 10 siderophore-producing bacteria were identified using the selective agar assay, displaying siderophore unit (SU) values ranging from 11.90% to 80.39%. It is noteworthy that *Erwinia* sp. QZ-E9 exhibited the highest siderophore production capacity, achieving an SU of 80.39%. A microcosm co-cultivation experiment was conducted with the strain QZ-E9 in iron-deficient conditions (2 μmol/L Fe^3^⁺). The results demonstrated that strain QZ-E9 significantly enhanced the growth of *S. portulacastrum*, by increases in both fresh weight (1.41 g) and root length (18.7 cm). Furthermore, fluorescence in situ hybridization (FISH) was utilized to ascertain the colonization pattern of strain QZ-E9 within the plant roots. The analysis demonstrated that strain QZ-E9 exhibited extensive colonization of the epidermal and outer cortical cells of *S. portulacastrum* roots, as well as the intercellular spaces and vascular tissues. This colonization indicated that *Erwinia* sp. QZ-E9 plays a crucial role in promoting the growth of *S. portulacastrum*, presumably through its siderophore-mediated iron acquisition mechanism.

## 1. Introduction

Phytoremediation refers to the use of plants and the metabolic activities of inter-root microbes to absorb, accumulate, degrade, or transform pollutants in contaminated environments, thereby purifying and restoring ecosystems [1]. However, the success of phytoremediation processes is often hindered by various factors, including limited nutrient availability, poor soil structure, and the toxicity of the contaminants themselves. Plant growth-promoting rhizobacteria (PGPR) play critical roles in enhancing the efficiency of phytoremediation. PGPR are a diverse group of bacteria that reside in the rhizosphere—the narrow zone surrounding plant roots—and promote plant growth and health through a variety of direct and indirect mechanisms such as nitrogen fixation, chelating metal ions, and producing ACC deaminase, etc. [2]. Among the diverse mechanisms employed by PGPR, siderophore-producing bacteria contribute significantly to phytoremediation efficiency. In the process of phytoremediation, siderophore-producing PGPR facilitate iron uptake by plants, thereby enhancing their metabolic activities and stress tolerance. Iron is essential for the growth of most microorganisms, serving crucial roles in enzyme activities and electron transfer. However, iron availability is often limited in marine environments due to its oxidation and subsequent insolubility. To address this, microorganisms produce siderophores—small, high-affinity iron-chelating compounds that enhance iron solubility from organic complexes, thereby improving iron bioavailability [3]. During phytoremediation, siderophore-producing bacteria play a critical role in overcoming iron deficiency stress in marine environments, meeting their own growth requirements while indirectly promoting plant growth by chelating iron ions [4]. Therefore, studying siderophore-producing bacteria is essential for enhancing the efficiency of phytoremediation.

A number of PGPR have been isolated as siderophore-producing bacteria, many of which belong to *Bacillus*, *Pseudomonas*, *Burkholderia*, and *Streptomyces*, and have been proven to have the ability to promote plant growth [5,6]. However, current research on the effectiveness of PGPR in promoting bioremediation mostly focuses on phytoremediation in soil environments and freshwater environments, while relatively few studies have been conducted on phytoremediation in marine environments.

*Sesuvium portulacastrum* is a halophytic plant with the proven ability to remove nitrogen, phosphorus, heavy metals, and organic pollutants. It is therefore widely used in aquatic phytoremediation [7,8,9]. Despite the successful application of *S. portulacastrum* for aquatic phytoremediation through ecological floating bed technology, there is still much to learn about the rhizosphere microorganisms. This study isolated endophytic bacteria from the roots of *S. portulacastrum* and conducted further research on the characteristics of siderophore-producing bacteria among them. It also explored their growth-promoting effects on *S. portulacastrum*. The results provide theoretical support and practical guidance for *S. portulacastrum* phytoremediation.

## 2. Results

### 2.1. Isolation of and Identification of Endophytes

**Figure 1:**
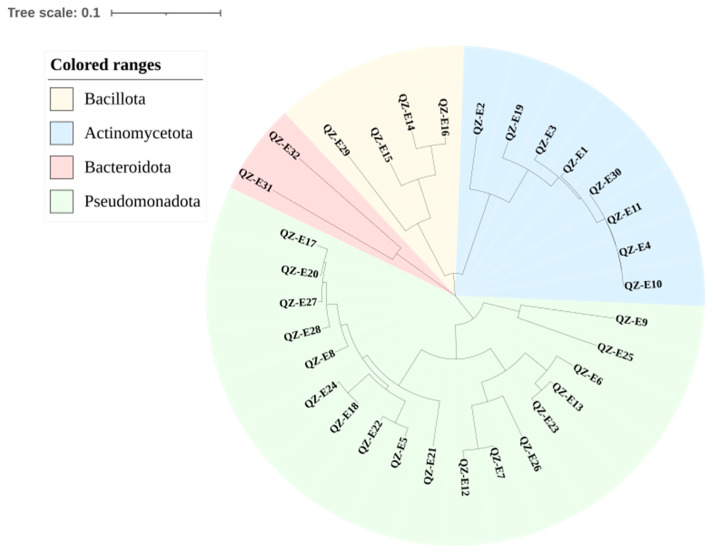
Phylogenetic analysis of isolated bacteria based on 16S rRNA gene sequences.

A total of 33 strains of endophytes were isolated, which belonged to 16 genera and 26 species after 16S rRNA gene sequence analysis. The sequences of the strains were aligned with EzBiocloud (www.ezbiocloud.net), and the results are shown in Table 1. We conducted a phylogenetic analysis using the Mega7.0 software with the neighbor-joining method, and the results are shown in Figure 1. The strains were identified as belonging to the phyla Actinomycetota, Bacteroidota, Bacillota, and Pseudomonadota. In 2016, Zhao et al. [10] studied, for the first time, the community structure of symbiotic bacteria within the root system of the halophyte *Salicornia europaea* at different growth stages by 454 high-throughput sequencing. Their findings revealed that Proteobacteria dominated the rhizosphere, followed by Bacteroidetes, Actinobacteria, and Firmicutes, which align with the results of this study. 

### 2.2. Screening of Siderophore-Producing Bacteria and Results of Activity Assay

**Figure 2:**
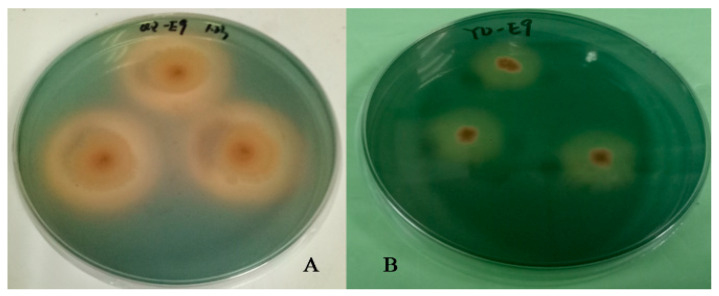
Siderophore-producing bacteria determined by CAS agar plate. ((**A**): Strain QZ-E9; (**B**): Strain QZ-E23).

The CAS plate assay results clearly showed that the strains forming orange transparent circles on the plate were the ones that had the ability to produce siderophores. The results of the assay are shown in Figure 2. The strains with positive plate assays were subjected to CAS liquid assays, and their siderophore-producing abilities were determined and expressed as siderophore units (SUs). Strains with SU values higher than 50% were identified as high siderophore-producing bacteria. The CAS plate assay results for siderophore-producing bacteria are presented in Table 2. Table 2 shows the strains with siderophore production ability and SU values that were selected for further study. This study screened 10 strains of siderophore-producing bacteria, with siderophore units ranging from 10.39% to 80.39%. QZ-E9 had the strongest ability to produce siderophores, with SUs reaching over 80%. The siderophore-producing bacteria were identified as belonging to the genera *Roseibium*, *Erwinia*, *Rossellomorea*, *Qipengyuania*, *Microbacterium*, and *Algoriphagus*.

### 2.3. Relationship between Siderophore-Producing Activity and Iron Ion Concentration in the Siderophore-Producing High-Yielding Bacterium Erwinia sp. QZ-E9

**Figure 3:**
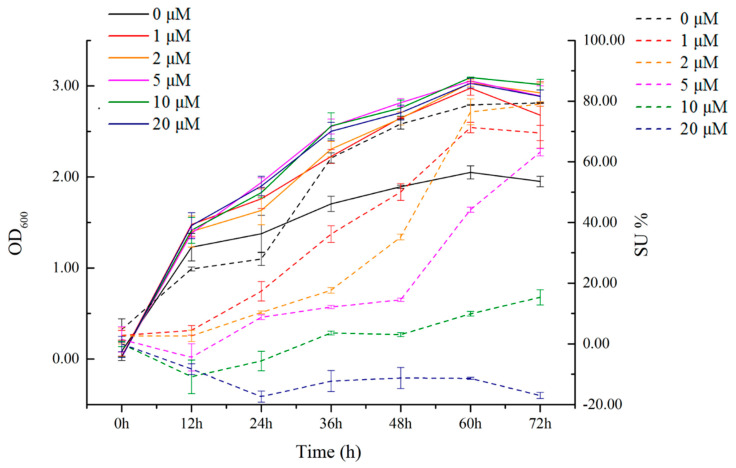
OD_600_ and SU values of *Erwinia* sp. QZ-E9 in different iron ion concentrations. The solid line is the change in the OD_600_ value of the strain under different addition groups, and the dotted line is the change in SU value under different addition groups.

Figure 3 shows the growth and SU values of strain QZ-E9 under different iron ion concentration conditions. The OD_600_ values of the different experimental groups clearly demonstrate that the growth rate of the strain is directly correlated with the concentration of iron ions. When the concentration of iron ions was 0 μmol/L, the OD_600_ value was significantly lower than that of the other added groups. The OD_600_ values of the other groups reached close to 3.0, while the group with no iron ions reached only 2.0 in 60 h. This proves that the growth rate and biomass of the strain were clearly slower than those of the iron ion added groups when the concentration of iron ions was 0 μmol/L. The SU value of the strain reached 80% at 72 h and 0 μmol/L, which was significantly higher than that of the iron ion addition group. Similarly, at 0 μmol/L, the SU value of the strain was much higher than that of the other experimental groups, indicating that the iron-producing capacity of the strain remained high at this time.

The SU was highest under the condition of no addition of iron ions, reaching 79.4%. Although no additional iron ions were added to the culture medium, the iron chelated by the bacteria may have originated from trace amounts of iron contained in the water used to prepare the culture medium. However, as the concentration of iron ions increased, the SU value decreased, its production rate decreased, and the time to reach stability increased. From the figure, it can be observed that when the concentration of iron ions reached 5 μmol/L, the strain still had high siderophore-producing ability. When the concentration was increased to 10 μmol/L, the SU value decreased significantly and even became negative at 20 μmol/L. This result indicates that strain QZ-E9 does not experience further iron deficiency stress when the concentration of iron ions exceeds 10 μmol/L.

### 2.4. Results of Simulation Experiments on Root Regression of Plant-Promoting Bacteria

#### 2.4.1. Effect of *Erwinia* sp. QZ-E9 on the Growth of *S. portulacastrum*

Through the above experiments, we screened to obtain the siderophore high-yielding bacterial strain QZ-E9, with 80.39% siderophore units, and compared its 16S rRNA gene sequence on the EzBiocloud culturable database. The similarity between strain QZ-E9 and *Erwinia rhpontici* ATCC 29283^(T)^ was highest, reaching 98.67%; consequently, it was named *Erwinia* sp. QZ-E9.

**Figure 4:**
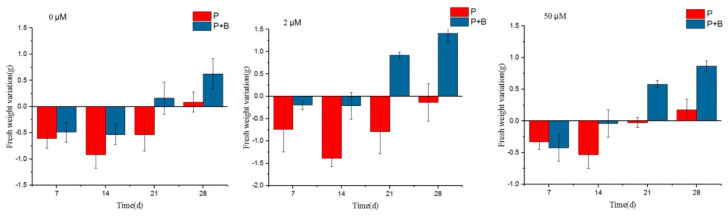
Fresh weight variations of *S. portulacastrum* under different treatments.

**Figure 5:**
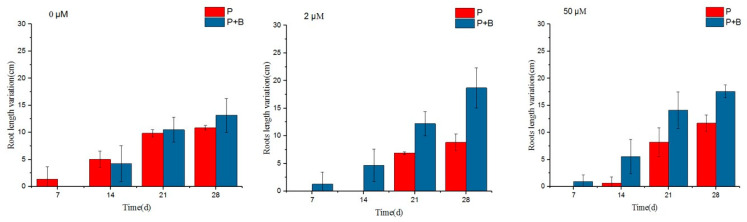
Root length variations of *S. portulacastrum* under different treatments.

To investigate the growth-promoting effect of the siderophore-producing bacterium *Erwinia* sp. QZ-E9 on *S. portulacastrum*, the fresh weight, plant height, leaf length, root length, and number of roots of *S. portulacastrum* in each experimental group were measured during the experiment as a measure of growth, and the results are shown in Figure 4 and Figure 5.

**Figure 6:**
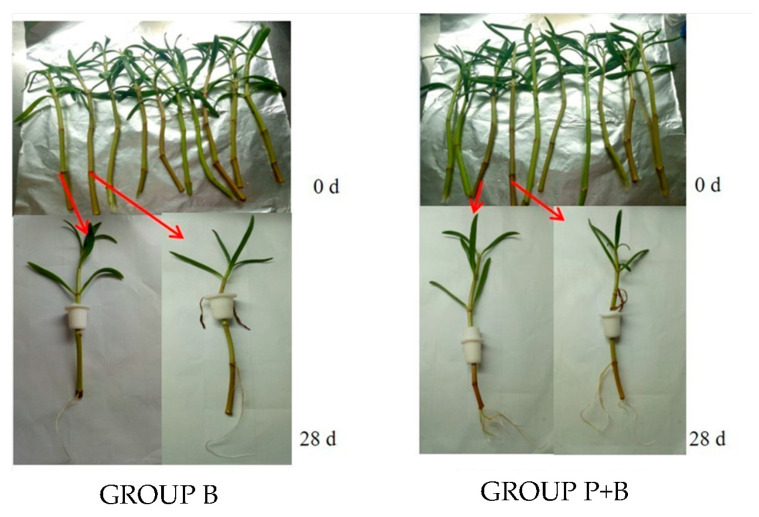
*S. portulacastrum* inoculated with *Erwinia* sp. QZ-E9 in 0 μmol/L iron ion.

Figure 6 shows the growth of *S. portulacastrum* in the P and P + B groups inoculated with *Erwinia* sp. QZ-E9 at a concentration of 0 μmol/L of Fe ions over time. Since the growth parameters of *S. portulacastrum* under different iron ion concentration conditions will be analyzed in detail in Figure 4 and Figure 5, only the iron ion concentration of 0 μmol/L is used as an example to show the changes in *S. portulacastrum* during the whole growth process. From the figure, it can be seen that the root morphologies of the P + B group and the P group were different; the number of main roots and lateral roots of the plants in the P + B group was higher, the root was more developed than that of the P group, and the surface area of the plant root was larger, which was convenient for the strains to be adsorbed to it.

Figure 4 shows the changes in the fresh weight of *S. portulacastrum* under different treatment conditions. It can be seen that the fresh weight of group P was lower than the starting value in the first 21 d, and both reached the lowest value at 14 d and then began to grow slowly. The maximum fresh weight growth at the end of the experiment was at the iron ion concentration of 50 μmol/L, with an increment of 0.17 g. At different iron ion concentrations, the net growth of group P + B started from the beginning of the 21 d, and at the end of the experiment, the maximum net growth occurred at the iron ion concentration of 2 μmol/L, with an increment of 1.41 g. Correlation analysis revealed that the maximum growth occurred at the iron ion concentration of 2 μmol/L, with an increment of 1.41 g. At the end of the experiment, the maximum net weight increase was 1.41 g at an iron ion concentration of 2 μmol/L. Correlation analysis showed that at an iron ion concentration of 2 μmol/L, there was a significant difference in the fresh weight of *S. portulacastrum* in the P + B group inoculated with QZ-E9 at the end of the experiment as compared with the control P group, which indicated that the strain had a significant role in increasing the fresh weight of *S. portulacastrum*.

Figure 5 and Table 3 reflect the changes in root length and number of roots of *Sesuvium portulacastrum* during the experiment, respectively. From Figure 5, it can be seen that at the iron ion concentration of 0 μmol/L, the growth of the P + B group inoculated with *Erwinia* sp. QZ-E9 was similar to that of the P group, and the root lengths at the end of the experiment were 13.17 cm and 10.87 cm, respectively; at the iron ion concentration of 0 μmol/L, the number of roots in the P + B group increased compared with that of the P group, and finally, the P + B group reached 4 strips/plant, while the P group reached only 1 strip/plant. At 2 μmol/L and 50 μmol/L, the growth of the corresponding P + B and P groups was similar, and at 28 d, the root lengths of the P + B group were 18.7 cm and 17.6 cm, and the numbers of *S. portulacastrum* were 7 and 4/plant, respectively, while the root lengths of the P group were 8.83 cm and 11.7 cm, and the numbers of *S. portulacastrum* were 3 and 1/plant, respectively. The number of *S. portulacastrum* roots was 3 and 1/plant, respectively. It was found that the root length of the P + B group was significantly higher than that of the B group under iron-poor (2 μmol/L) conditions. In conclusion, *Erwinia* sp. QZ-E9 can better promote the growth of *S. portulacastrum* fresh weight and root under Fe-depleted conditions.

#### 2.4.2. *Erwinia* sp. QZ-E9 Colonization in *S. portulacastrum*

**Figure 7:**
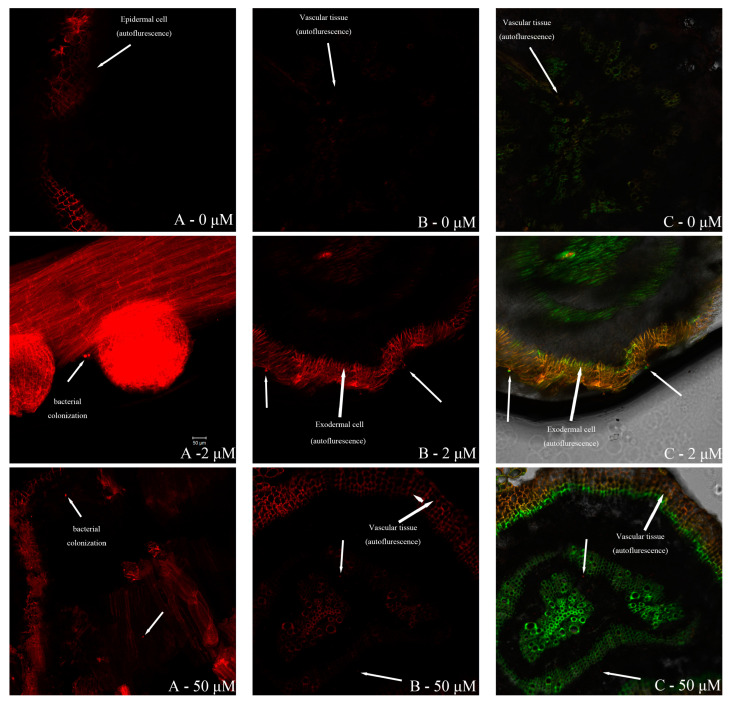
FISH images inside the roots of *S. portulacastrum* under different treatments ((**A**) shows the excited image of the EUB338 probe labeled with Cy3 only in group P; (**B**) shows the excited image of the EUB338 probe labeled with red fluorescent Cy3 in group P + B; and (**C**) shows the excited image of the probe labeled with Cy3 and the probe labeled with green fluorescent FITC at the same time in group P + B. The magnification is 10× and all the lengths of the scales in the figure are 50 μmol/L).

Figure 7 shows the FISH images inside the roots of *S. portulacastrum* under different treatments. The red fluorescence is the EUB338 probe labeled with Cy3 [11], and the green fluorescence is the EBAC1790 probe labeled with FITC [12] used for the detection of strain QZ-E9.

By contrasting the colonization patterns of *Erwinia* sp. QZ-E9 within *S. portulacastrum* roots at different iron ion concentrations, we observed weaker fluorescence at 0 μmol/L compared to 2 μmol/L and 50 μmol/L. This suggests reduced colonization in the absence of iron ions. Conversely, similar fluorescence intensities were observed at 2 μmol/L and 50 μmol/L, indicating comparable levels of colonization. By observing the location of the green fluorescence, it was found that *Erwinia* sp. QZ-E9 could colonize the epidermis, the outer cortex cells, and the cellular interstitial space of both, as well as the vascular tissues of the rhizosphere of *S. portulacastrum*.

## 3. Discussion

This study explores the role of siderophore-producing endophytic bacteria in promoting the growth of *S. portulacastrum*, a halophyte known for its stress tolerance and potential applications in challenging environments. While phytoremediation was mentioned as a potential application in the introduction, this study primarily focused on the growth-enhancing properties of these bacteria rather than directly measuring indicators of phytoremediation, such as heavy metal uptake or soil detoxification.

### 3.1. Endophytic Bacteria and Plant Growth Promotion

Our findings demonstrated significant growth promotion in *S. portulacastrum* when inoculated with siderophore-producing bacteria. This is consistent with a growing body of research on the role of endophytes in enhancing plant growth under stress conditions [13]. Endophytic bacteria have been shown to enhance plant growth by producing phytohormones, solubilizing nutrients, and improving stress tolerance [14]. Specifically, the production of siderophores by these bacteria plays a key role in facilitating iron uptake in iron-limited soils, which is crucial for plant development [15]. 

Recent studies have further highlighted the complex interactions between endophytes and their host plants, particularly in stress environments. For example, Zhang et al. demonstrated that endophytic bacteria could modulate plant metabolic pathways, leading to improved tolerance to both abiotic and biotic stresses [16]. This suggests that the growth promotion observed in our study could be part of a broader systemic response induced by these beneficial microbes.

Moreover, the integration of endophytes into sustainable agriculture practices is becoming an increasingly attractive area of research. The use of plant growth-promoting bacteria (PGPB) is being explored as a biological alternative to chemical fertilizers and pesticides, with the potential to reduce environmental impact and improve crop yields under adverse conditions [17]. This aligns with our findings that siderophore-producing bacteria can significantly enhance the growth of *S. portulacastrum*, suggesting that these microbes could play a role in both ecological restoration and sustainable agriculture.

### 3.2. Siderophores and Heavy Metal Stress Alleviation

Siderophores not only aid in iron acquisition but also play a crucial role in mitigating heavy metal toxicity in plants. Research has shown that siderophores can chelate toxic metals such as cadmium, lead, and chromium, thereby reducing their bioavailability and toxic effects on plants [18]. This dual function of siderophores—facilitating essential nutrient uptake and detoxifying heavy metals—has significant implications for plant growth in contaminated soils [6].

Our study supports the hypothesis that siderophore-producing bacteria can enhance plant growth by mitigating heavy metal stress, even though we did not directly measure heavy metal uptake. Recent studies by Dimkpa et al. and Kim et al. have highlighted the potential of siderophore-producing endophytes in bioremediation efforts, particularly in plants growing in metal-contaminated environments [19,20]. These studies suggest that the interactions between endophytes and plants may not only promote growth but also contribute to the plant’s ability to tolerate and accumulate heavy metals.

### 3.3. Potential Applications in Phytoremediation

Although this study did not focus on phytoremediation metrics, the results indicate that *S. portulacastrum* inoculated with siderophore-producing bacteria has the potential to be used in phytoremediation strategies. Future studies should aim to measure both growth promotion and phytoremediation outcomes, such as metal uptake in plant tissues, soil metal concentrations, and the plant’s overall detoxification capacity.

The potential for combining microbial inoculants with plants for bioremediation has been gaining traction in recent years. Recent studies by Zhao et al. (2024) and Qiu et al. (2024) have emphasized the synergistic effects of plant–microbe partnerships in enhancing both plant health and environmental remediation efforts [21,22]. These studies underscore the need for integrative approaches that consider both plant growth and contaminant removal when assessing the effectiveness of phytoremediation strategies.

To realize the full potential of *S. portulacastrum* in phytoremediation, future research should include detailed investigations into the mechanisms by which endophytic bacteria influence metal uptake, translocation, and detoxification in plants. Studies should also explore the long-term sustainability of using microbial inoculants in real-world contaminated sites, as well as the ecological impacts of these interventions.

## 4. Materials and Method

### 4.1. Plant Material and Bacterial Strains

Root samples of *S. portulacastrum* were collected from the base of plants located in Quanzhou Bay, China (118°39′18.6″ E, 24°54′32.7″ N), on 23 March 2016. Samples were placed in a 4 °C ice box immediately after collection and transported back to the laboratory for subsequent isolation of symbiotic bacteria within the rhizosphere of *S. portulacastrum*.

### 4.2. Root Treatment and Isolation of Endophytic Bacteria in S. portulacastrum

The collected *S. portulacastrum* root samples were rinsed in sterile water for 3–5 min after removing the delicate fibrous roots to remove surface impurities and soil. The root samples were cut into small sections of 3–5 cm using sterile scissors, and 1.00 g of fresh root sample was accurately weighed and set aside after absorbing the surface moisture. First, the samples were immersed in 75% ethanol solution and shaken at 180 rpm/min for 3 min; subsequently, they were immersed in 2.5% sodium hypochlorite solution and shaken under the same conditions for 5 min; finally, they were immersed again in 75% ethanol solution and shaken for 30 s. Finally, the root was rinsed three times with sterile water. A 100 μL volume of the last rinsing solution was spread on a 2216E plate and incubated at 28 °C for 3–5 days. If no colonies were formed after incubation, surface disinfection of the roots was considered complete. The fully sterilized sample was ground in a sterilized mortar, 9 mL of sterile water was added for gradient dilution and shaken well, then 100 μL of the supernatant solution was taken and spread on the 2216E plate, which was then incubated at 28 °C until the formation of colonies. According to the morphological characteristics of the colonies, different single colonies were selected for delineation and further purification. 16S rRNA sequence analysis was performed on the isolated and purified strains, and a series of endophytic bacteria within the *S. portulacastrum* were finally isolated. The isolated strains were stored in 40% (*v*/*v*) glycerol suspension at −80 °C.

### 4.3. Screening and Activity Determination of Siderophore-Producing Bacteria

The CAS plate assay [23] was used to preliminarily identify whether the strains had the ability to produce siderophores. The isolated strains were inoculated on CAS plates and incubated at 28 °C for 48 h. The strains with siderophore-producing ability were initially screened out by observing whether there was an orange transparent circle and the transparent circle size. CAS liquid assay was used to determine the siderophore-producing activity of the strains [24]. The purified strains were inoculated into 2216E broth, incubated at 28 °C at 180 r/min for 24 h, and centrifuged at 5000 r/min for 15 min to collect the bacterial bodies, which were washed three times with MKB broth. The concentration of the bacterial solution was adjusted to 10^6^ cells/mL and then inoculated into MKB broth at an inoculation rate of 1%, incubated at 28 °C at 180 r/min for 48 h, and centrifuged at 5000 r/min for 15 min to collect the bacterial bodies. The samples were incubated at 180 r/min at 28 °C for 48 h. After centrifugation at 8000 r/min for 15 min, 3 mL of supernatant was taken and an equal volume of CAS solution was added and shaken well for 1 h. The color change of the liquid was observed, and the absorbance value (As) of each sample was measured at 630 nm. In the control group, uninoculated MKB broth was used, and OD_630_ (Ar) was measured after the same treatment. The siderophore-producing ability of the strains was expressed as siderophore units (SUs), calculated as SU = [(Ar − As)/Ar] × 100%, with three parallels for each sample.

### 4.4. Method for Determining the Relationship between Siderophore-Producing Activity and Iron Ion Concentration in the Siderophore-Producing Bacterium Strain QZ-E9

Strain QZ-E9 was inoculated into 2216E broth, incubated at 28 °C for 24 h at 180 r/min, and then centrifuged at 5000 r/min for 15 min to collect the bacterial bodies, which were washed three times with MKB medium. The original cell number was adjusted to 10^6^ cells/mL and inoculated into 50 mL of MKB medium containing different concentrations of iron ions (0, 1, 2, 5, 10, 20 μmol/L). The culture medium was incubated at 180 r/min at 28 °C for 72 h. During this period, 2 mL of the bacterial fluid was taken for each 12 h for the determination of bacterial concentration and siderophore units. The bacterial concentration was expressed by OD600, and the siderophore units were determined by CAS liquid assay, with three parallels in each group.

### 4.5. Simulation Experiment on Root Regression of Plant-Promoting Bacteria

Plant samples for the experiment were selected from 70 well-grown *S. portulacastrum* plants precultured for 2 months in Hoagland nutrient solution (Table 4) at a salinity of 20, where the *S. portulacastrum* was retained with four stem nodes, six leaves, and a fresh weight of approximately 4.5 g per plant. Eighty-two 100 mL sterilized flasks were prepared prior to the experiment, 100 mL of sterilized Hoagland nutrient solution was added to each flask, and the flasks were wrapped in aluminum foil to prevent algal blooms inside the flasks and the effect of light on the plant roots. Before the experiment, the stems of the *S. portulacastrum* used were sterilized by immersing them in an effective chlorine solution of 2% sodium hypochlorite for 5 min, followed by washing them three times with sterile water to sterilize their surfaces.

In order to understand the effect of plant growth-promoting bacteria obtained from screening on the growth of *S. portulacastrum*, three groups were set up: bacterial group (B), plant group (P), and plant + bacteria group (P + B); three replicates were set up for group B, 10 replicates for each of groups P and P + B, and one surface-sterilized *S. portulacastrum* was placed into each flask of groups P and P + B. Three different iron concentrations of 0 μmol/L (iron-free), 2 μmol/L (iron-poor), and 50 μmol/L (iron-rich) were set for group B, group P, and group P + B inoculated with the iron-carrier-producing bacterium QZ-E9 (9.5 × 10^8^ cells/mL), and the plants were cultured at 25 °C for 28 d under alternating conditions of 14 h of light (3500 lx) and 10 h of darkness.

During the experiment, 6 mL of culture solutions from groups B and P + B were collected on days 0, 7, 14, 21, and 28, of which 5 mL was used for DNA extraction as a qPCR template to quantify the microbial content in the culture system, and the other 1 mL was counted by the MPN method to determine the abundance of the added strains. Fresh weight, plant height, leaf length, root length, and number of roots were measured during the experiment. After 28 d of the experiment, the root systems of *S. portulacastrum* in the different experimental groups were collected, manually sliced, and fixed for FISH experiments [25] to determine the colonization of *S. portulacastrum* by the added bacteria and to combine the parameters with the above parameters to determine the contribution of the added bacteria to the growth of *S. portulacastrum*.

### 4.6. Detection of Bacterial Colonization

Fluorescence in situ hybridization was used to observe the colonization of growth-promoting endosymbiotic bacteria inoculated in the culture system of *S. portulacastrum*, and the experimental method was as referred to by Kliot et al. [18]. The root of *S. portulacastrum* at the end of the 28 d experiment was taken and manually sliced using a sharp blade. The thinly sliced tissues were placed into 1.5 mL sterilized centrifuge tubes and Carnoy’s fixative (chloroform/ethanol/glacial acetic acid = 6:3:1, *vol.*/*vol.*) added to fix them overnight at room temperature; fixed samples can be stored in anhydrous ethanol for a few days to a few weeks. The fixed plant tissue sections were taken and washed with hybridization buffer (20 mM Tris-HCl, pH 8.0; 0.9 M NaCl; 0.01% (*wt.*/*vol.*/*vol.*) SDS; 20% (*vol.*/*vol.*) formamide) three times for 1 min each time. A 500 μL volume of hybridization buffer was added to the washed tissue sections, the corresponding probes were then added to a final concentration of 10 pmol/mL, and the samples placed in a dark environment and hybridized overnight at room temperature. The sectioned tissues after hybridization were washed three times with hybridization buffer for 1 min each time. The treated plant tissue sections, placed on slides covered with coverslips, sealed with nail polish, and wrapped with aluminum foil, were examined microscopically under a confocal fluorescence microscope.

## 5. Conclusions

In this study, a total of 33 strains of endophytic bacteria were isolated based on the culturable method in this investigation, which belonged to 13 genera and 25 species according to the 16S rRNA gene sequence analysis. A total of 10 strains of siderophore-producing bacteria were screened by the CAS plate assay, and the siderophore units of the strains were determined by the CAS assay liquid method. The results of the SU assay ranged from 11.90% to 80.39%, among which strain *Erwinia* sp. QZ-E9 had the highest siderophore-producing activity at 80.39%. The plant growth-promoting ability of strain *Erwinia* sp. QZ-E9 was evaluated by a microcosm co-cultivation experiment, and the results showed that *Erwinia* sp. QZ-E9 had a significant growth-promoting effect on the growth of *S. portulacastrum* fresh weight and root length under iron-poor conditions, with a final increment of 1.41 g and 18.7 cm, respectively. Using FISH technology to observe the distribution of bacterial colonization in the root system of *S. portulacastrum*, it was found that *Erwinia* sp. QZ-E9 was able to colonize the epidermis, the outer cortex cells, the intercellular space of both, and the vascular tissues of the root system of *S. portulacastrum*. In conclusion, the siderophore-producing bacterium *Erwinia* sp. QZ-E9 had a beneficial impact on the growth of *S. portulacastrum*. Therefore, it has a certain potential in improving the efficiency of phytoremediation.

## Figures and Tables

**Table 1:** Identification based on 16S rRNA sequence of endophytic bacteria isolated from halophyte *S. portulacastrum*.

Strains	Reference Bacteria of Highest Similarities	Similarities
QZ-E1	*Microbacterium pumilum* KV-488^(T)^	98.96
QZ-E2	*Agromyces kandeliae* Q22^(T)^	98.89
QZ-E3	*Microbacterium fluvii* YSL3-15^(T)^	99.36
QZ-E4	*Microbacterium pumilum* KV-488^(T)^	97.70
QZ-E5	*Auramtiacibacter aquimixticola* JSSK-14^(T)^	97.55
QZ-E6	*Roseibium litorale* 4C16A^(T)^	99.70
QZ-E7	*Devosia naphthalenivorans* CM5-1^(T)^	97.84
QE-E8	*Qipengyuania citrea* RE35F/1^(T)^	97.77
QZ-E9	*Erwinia rhpontici* ATCC 29283^(T)^	98.67
QZ-E10	*Microbacterium schleiferi* IFO 15075^(T)^	99.15
QZ-E11	*Microbacterium schleiferi* IFO 15075^(T)^	98.65
QZ-E12	*Devosia naphthalenivorans* CM5-1^(T)^	97.05
QZ-E13	*Roseibium aggregatum* IAM 12614^(T)^	98.99
QZ-E14	*Rossellomorea oryzaecorticis* R1^(T)^	98.25
QZ-E15	*Alkalihalobacillues algicola* KMM 3737^(T)^	99.10
QZ-E16	*Rossellomorea marisflavi* TF-11^(T)^	99.11
QZ-E17	*Qipengyuania polymorpha* 1NDH17^(T)^	98.56
QZ-E18	*Alterricroceibacterium indicum* MSSRF26^(T)^	99.93
QZ-E19	*Microbacterium testaceum* NBRC 12675^(T)^	98.31
QZ-E20	*Qipengyuania polymorpha* 1NDH17^(T)^	99.06
QZ-E21	*Parasphingorhabdus pacifica* KMM9574^(T)^	98.20
QZ-E22	*Aurantiacibacter arachoides* RC4-10-4^(T)^	98.84
QZ-E23	*Roseibium aggregatum* IAM 12614^(T)^	98.84
QZ-E24	*Alterricroceibacterium indicum* MSSRF26^(T)^	99.93
QZ-E25	*Marinomonas primoryensis* KMM3633^(T)^	97.59
QZ-E26	*Devosia humi* THG-MM1^(T)^	95.49
QZ-E27	*Qipengyuania vulgaris* 022 2-10^(T)^	99.93
QZ-E28	*Qipengyuania xiapuensis* 1NDW9^(T)^	98.27
QZ-E29	*Lactococcus lactis* subsp. lactis JCM 5805^(T)^	98.49
QZ-E30	*Microbacterium schleiferi* IFO 15075^(T)^	99.22
QZ-E31	*Algoriphagus halophilus* DSM 15292^(T)^	98.66
QZ-E32	*Aestuariibaculum suncheonense* SC17^(T)^	97.94
QZ-E33	*Erythrobacter westpacificensis* JLT 2008^(T)^	97.57

**Table 2:** The SU values of high-yield siderophore-producing bacteria.

Strains	Genus	SU%	SU Activity
QZ-E6	*Roseibium*	27.51	++
QZ-E9	*Erwinia*	80.39	+++++
QZ-E13	*Roseibium*	48.59	+++
QZ-E14	*Rossellomorea*	59.51	+++
QZ-E16	*Rossellomorea*	29.66	++
QZ-E17	*Qipengyuania*	14.00	+
QZ-E19	*Microbacterium*	13.90	+
QZ-E20	*Qipengyuania*	13.03	+
QZ-E23	*Roseibium*	11.90	+
QZ-E31	*Algoriphagus*	20.99	++

SU size is indicated by + for 10–20% siderophore-producing activity; 20–40%: ++; 40–60%: +++; 60–80%: ++++; 80–100%: +++++.

**Table 3:** Root number variations in *S. portulacastrum*.

Time	QZ-E9 (0 μmol/L)		QZ-E9 (2 μmol/L)		QZ-E9 (50 μmol/L)	
	P	P + B	P	P + B	P	P + B
7 d	0	0	0	0	0	2
14 d	1	2	0	2	0	2
21 d	1	3	2	6	1	4
28 d	1	4	3	7	1	4

**Table 4:** The Hoagland nutrient solution used in the experiment.

Major Element Nutrient Solution		Trace Element Nutrient Solution	
Formulation	Concentration 1.0 × 10^−3^ mol/L	Formulation	Concentration 1.0 × 10^−6^ mol/L
Ca(NO_3_)·4H_2_O	2.0	H_3_BO_3_	1.0
K_2_SO_4_	0.75	MnSO_4_·H_2_O	1.0
KCl	0.10	ZnSO_4_·7H_2_O	1.0
KH_2_PO_4_	0.25	CuSO_4_·5H_2_O	0.10
MgSO_4_·7H_2_O	0.65	(NH_4_)MoO_4_·4H_2_O	5.0 × 10^−3^

## Data Availability

The 16S rRNA sequence data of 33 isolated endophytic bacteria were deposited under GenBank Nos. PQ047699-PQ047731.

## References

[1] Sam Cherian S.L., Nele W., Vangronsveld J. (2012). Phytoremediation of Trace Element–Contaminated Environments and the Potential of Endophytic Bacteria for Improving This Process. Crit. Rev. Environ. Sci. Technol..

[2] Laller R., Khosla P.K., Negi N., Avinash H., Kusum, Thakur N., Kashyap S., Shukla S.K., Hussain I. (2023). Bacterial Volatiles as PGPRs: Inducing Plant Defense Mechanisms during Stress Periods. S. Afr. J. Bot..

[3] Saha M., Sarkar S., Sarkar B., Sharma B.K., Bhattacharjee S., Tribedi P. (2016). Microbial Siderophores and Their Potential Applications: A Review. Environ. Sci. Pollut. Res..

[4] Sujatha N.T., Ammani K. (2013). Siderophore Production by the Isolates of Fluorescent Pseudomonads. Int. J. Curr. Res. Rev..

[5] Sun Y., Wu J., Shang X., Xue L., Ji G., Chang S., Niu J., Emaneghemi B. (2022). Screening of Siderophore-Producing Bacteria and Their Effects on Promoting the Growth of Plants. Curr. Microbiol..

[6] Sultana S., Alam S., Karim M.M. (2021). Screening of Siderophore-Producing Salt-Tolerant Rhizobacteria Suitable for Supporting Plant Growth in Saline Soils with Iron Limitation. J. Agric. Food Res..

[7] Lokhande V.H., Nikam T.D., Suprasanna P. (2009). *Sesuvium portulacastrum* (L.) L. a Promising Halophyte: Cultivation, Utilization and Distribution in India. Genet. Resour. Crop Evol..

[8] Fourati E., Wali M., Vogel-Mikuš K., Abdelly C., Ghnaya T. (2016). Nickel Tolerance, Accumulation and Subcellular Distribution in the Halophytes *Sesuvium portulacastrum* and *Cakile maritima*. Plant Physiol. Biochem..

[9] Lokhande V.H., Patade V.Y., Srivastava S., Suprasanna P., Shrivastava M., Awasthi G. (2020). Copper Accumulation and Biochemical Responses of *Sesuvium portulacastrum* (L.). Mater. Today Proc..

[10] Zhao S., Zhou N., Zhao Z.-Y., Zhang K., Tian C.-Y. (2016). High-Throughput Sequencing Analysis of the Endophytic Bacterial Diversity and Dynamics in Roots of the Halophyte Salicornia Europaea. Curr. Microbiol..

[11] Amann R.I., Binder B.J., Olson R.J., Chisholm S.W., Devereux R., Stahl D.A. (1990). Combination of 16S rRNA-Targeted Oligonucleotide Probes with Flow Cytometry for Analyzing Mixed Microbial Populations. Appl. Environ. Microbiol..

[12] Bohnert J., Hübner B., Botzenhart K., Bohnert J. (2000). Rapid Identification of Enterobacteriaceae Using a Novel 23S rRNA-Targeted Oligonucleotide Probe. Int. J. Hyg. Environ. Health.

[13] Kong Z., Glick B.R., Poole R.K. (2017). Chapter Two—The Role of Plant Growth-Promoting Bacteria in Metal Phytoremediation. Advances in Microbial Physiology.

[14] Ahemad M., Kibret M. (2014). Mechanisms and Applications of Plant Growth Promoting Rhizobacteria: Current Perspective. J. King Saud Univ. Sci..

[15] Yi S., Wei M., Li F., Liu X., Fan Q., Lu H., Wu Y., Liu Y., Tian J., Zhang M. (2024). In-Situ Enrichment of ARGs and Their Carriers in Soil by Hydroxamate Siderophore: A Promising Biocontrol Approach for Source Reduction. Environ. Int..

[16] Li B., Wu B., Dong Y., Lin H., Liu C. (2023). Endophyte Inoculation Enhanced Microbial Metabolic Function in the Rhizosphere Benefiting Cadmium Phytoremediation by Phytolacca Acinosa. Chemosphere.

[17] Orozco-Mosqueda M.d.C., Glick B.R., Santoyo G. (2020). ACC Deaminase in Plant Growth-Promoting Bacteria (PGPB): An Efficient Mechanism to Counter Salt Stress in Crops. Microbiol. Res..

[18] Gomes A.F.R., Almeida M.C., Sousa E., Resende D.I.S.P. (2024). Siderophores and Metallophores: Metal Complexation Weapons to Fight Environmental Pollution. Sci. Total Environ..

[19] Kim D., Duckworth O.W., Strathmann T.J. (2009). Hydroxamate Siderophore-Promoted Reactions between Iron(II) and Nitroaromatic Groundwater Contaminants. Geochim. Cosmochim. Acta.

[20] Dimkpa C.O., Merten D., Svatoš A., Büchel G., Kothe E. (2009). Siderophores Mediate Reduced and Increased Uptake of Cadmium by Streptomyces Tendae F4 and Sunflower (*Helianthus annuus*), Respectively. J. Appl. Microbiol..

[21] Zhao Z., Liu L., Sun Y., Xie L., Liu S., Li M., Yu Q. (2024). Combined Microbe-Plant Remediation of Cadmium in Saline-Alkali Soil Assisted by Fungal Mycelium-Derived Biochar. Environ. Res..

[22] Qiu H., Xu J., Yuan Y., Alesi E.J., Liang X., Cao B. (2024). Low-Disturbance Land Remediation Using Vertical Groundwater Circulation Well Technology: The First Commercial Deployment in an Operational Chemical Plant. Sci. Total Environ..

[23] Schwyn B., Neilands J.B. (1987). Universal Chemical Assay for the Detection and Determination of Siderophores. Anal. Biochem..

[24] Kumar P., Thakur S., Dhingra G.K., Singh A., Pal M.K., Harshvardhan K., Dubey R.C., Maheshwari D.K. (2018). Inoculation of Siderophore Producing Rhizobacteria and Their Consortium for Growth Enhancement of Wheat Plant. Biocatal. Agric. Biotechnol..

[25] Kliot A., Ghanim M. (2016). Fluorescent in Situ Hybridization for the Localization of Viruses, Bacteria and Other Microorganisms in Insect and Plant Tissues. Methods.

